# Children and Young People With First Relapse or Progression of Upfront Metastatic Rhabdomyosarcoma: An Analysis of Clinical Features and Outcomes From the INternational Soft Tissue saRcoma ConsorTium (INSTRuCT)

**DOI:** 10.1002/cam4.71524

**Published:** 2026-02-20

**Authors:** Ajla T. Wasti, Gianni Bisogno, Beatrice Coppadoro, Ilaria Zanetti, Martin Ebinger, Amadeus T. Heinz, Henry C. Mandeville, Rita Alaggio, Michela Casanova, Sheila E. J. Terwisscha van Scheltinga, Rick R. van Rijn, Veronique Minard‐Colin, Daniel Orbach, Natalie B. Collins, Wei Xue, Rajkumar Venkatramani, Johannes H. M. Merks, Julia C. Chisholm

**Affiliations:** ^1^ Institute of Cancer Research Sutton UK; ^2^ Department of Women and Children's Health University of Padova Padova Italy; ^3^ Pediatric Hematology Oncology Division University Hospital of Padova Padova Italy; ^4^ Department of Pediatric Hematology and Oncology University Children's Hospital Tübingen Germany; ^5^ Stuttgart Cancer Center, Zentrum für Kinder‐, Jugend‐ und Frauenmedizin (Olgahospital), Pädiatrie 5 (Pädiatrische Onkologie, Hämatologie, Immunologie) Klinikum der Landeshauptstadt Stuttgart Stuttgart Germany; ^6^ Children and Young People's Unit Royal Marsden Hospital Sutton UK; ^7^ Istituto di Ricovero e Cura a Carattere Scientifico Bambino Gesù Children's Hospital Rome Italy; ^8^ Department of Medico‐Surgical Sciences and Biotechnologies Università Sapienza Rome Italy; ^9^ Fondazione IRCCS Istituto Nazionale dei Tumori Milan Italy; ^10^ Princess Máxima Center for Pediatric Oncology Utrecht the Netherlands; ^11^ Department of Radiology and Nuclear Medicine University of Amsterdam, Amsterdam UMC Amsterdam the Netherlands; ^12^ Department of Pediatric and Adolescent Oncology, INSERM U1015, Gustave Roussy Université Paris‐Saclay Villejuif France; ^13^ SIREDO Oncology Center (Care, Innovation and Research for Children, Adolescents and Young Adults With Cancer) PSL University, Institut Curie Paris France; ^14^ Dana‐Farber/Boston Children's Cancer and Blood Disorders Center Harvard Medical School Boston Massachusetts USA; ^15^ Department of Biostatistics University of Florida Gainesville Florida USA; ^16^ Department of Pediatrics, Division of Hematology Oncology Baylor College of Medicine Houston Texas USA

**Keywords:** INSTRuCT, metastatic, prognostic factors, progression, relapse, rhabdomyosarcoma

## Abstract

**Introduction:**

We evaluated the survival rate/survivor characteristics following first progression/relapse of metastatic rhabdomyosarcoma (M1 RMS), using pooled European and US collaborative group data from the INternational Soft Tissue saRcoma ConsorTium (INSTRuCT).

**Methods:**

Patients with first diagnosis of M1 RMS aged 0–40 years were identified within the INSTRuCT database (Upfront Cohort; UC). The First Event Cohort (FEC) included UC patients with first event of disease progression/relapse. Clinical features and survival of FEC patients were described.

**Results:**

UC included 1095 eligible M1 RMS patients. 5‐year Overall and Event Free Survival were 32.0% (95% Confidence Interval (CI) 29.2–34.9) and 27.5% (95% CI 24.8–30.2) respectively. Median time to event was 13.9 months (range 1 day‐172.6 months). Among UC patients, 727 with first event of progression/relapse were included in FEC. 3‐year Overall Survival for FEC from first event was 8.0% (95% CI 6.1–10.2). Thirty‐four (4.7%) FEC patients were alive with > 3 years follow up (“disease free”) and 16 (2.2%) with < 3 years follow up. FEC patients alive > 3 years were significantly more likely than deceased FEC patients to have: younger age (*p* = 0.0031); no locoregional lymph node involvement (*p* = 0.0013); fewer metastatic sites (*p* = 0.006); no bone and/or bone marrow disease (*p* < 0.001 for each); lower Oberlin scores (*p* < 0.0001); time to first event > 18 months (*p* < 0.0001). Univariate and multivariable analyses conducted in FEC to investigate factors impacting OS showed that Oberlin score ≥ 2 (Hazard Ratio (HR) 1.295, 95% Confidence Limits (CL) 1.07–1.57, *p* = 0.0074) and involvement of loco‐regional lymph nodes at diagnosis (HR 1.28, 95% CL 1.08–1.52, *p* = 0.0053) were associated with worse outcome.

**Conclusions:**

Outcomes following first progression/relapse of M1 RMS are dismal. Survivors had fewer adverse prognostic features at first presentation and later first events. Further work is required to predict survivors of first relapse more reliably.

## Graphical abstract

In this pooled analysis of 1095 patients with metastatic rhabdomyosarcoma, 3‐year overall survival was only 8% among the 727 patients with first event of relapse or progression. Patients “disease‐free” 3 years after first event had less locoregional lymph node involvement, fewer known adverse risk factors and lower Oberlin scores at first presentation than those who died and were more likely to have their first event > 18 months from diagnosis. In a multivariable Cox regression model stratified for time to relapse/progression, locoregional lymph node involvement and > 2 Oberlin risk factors at diagnosis were associated with worse outcome.

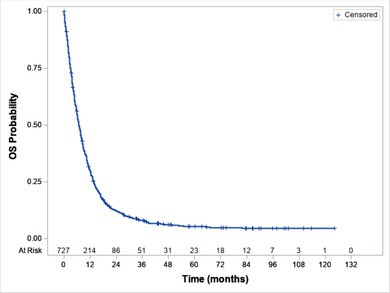

**Abbreviations**
AIEOP: Italian Association of Paediatric Haematology and Oncology
CI: confidence interval
CL: confidence limit
COG: Children's Oncology Group
CWS: Cooperative Weichteilsarkom Studiengruppe
EFS: event free survival
EpSSG: European paediatric Soft tissue sarcoma Study Group
FEC: First Event Cohort
FN: fusion negative
FNx: fusion status unknown
FP: fusion positive
GPOH: Gesellschaft für Pädiatrische Onkologie und Hämatologie
HR: hazard ratio
ICG: Italian Cancer Group
INSTRuCT: INternational Soft Tissue saRcoma ConsorTium
M1: metastatic
MMT: Malignant Mesenchymal Tumour study group
N0: node negative
N1: node positive
Nx: nodal status unknown
OS: overall survival
*PAX::FOXO1*
: paired box‐forkhead domain family member
R2: residual macroscopic disease
RMS: rhabdomyosarcoma
SIOP: International Society of Paediatric Oncology
T1: tumour confined to tissue of origin
T2: tumour extending beyond tissue of origin
Tx: tumour extent unknown
UC: Upfront Cohort

## 1 Introduction

Patient outcomes remain poor for metastatic rhabdomyosarcoma (M1 RMS), with recent clinical trials showing only marginal improvement in outcomes beyond a historical 3‐year event free survival (EFS) of 27% and overall survival (OS) of 34% [1]. The survival curves beyond 5 years demonstrate that salvage of relapsed M1 RMS is rare [1, 2, 3, 4] yet there are occasional patients where long‐term survival is achieved following a first event.

The widely used clinical risk factors (Oberlin factors) that predict for adverse EFS in M1 RMS, derived from a pooled analysis of 788 patients, are defined as follows: age < 1 or ≥ 10 years; bone or bone marrow metastasis; unfavourable site of primary disease (extremity or “other”) and 3 or more organ systems involved with metastasis [1]. In Oberlin's analysis, patients with zero risk factors had a 50% 3‐year EFS, reducing to only 5% if all 4 Oberlin risk factors were present. The study proposed 2 distinct prognostic groups: those with 0 or 1 risk factors comprised 42% of the patients and had a 3‐year EFS of 44%, compared to those with 2–4 risk factors who had a 3‐year EFS of only 14%. In the most recent European paediatric Soft tissue sarcoma Study Group (EpSSG) pooled analysis of 372 patients with metastatic RMS (EpSSG MTS 2008 and BERNIE studies) [3] a modification of these 2 prognostic groups was proposed combining 0–2 Oberlin risk factors (3‐year EFS 46.1%) and 3–4 Oberlin risk factors (3‐year EFS 12.5%; *p* < 0.0001).

Of note, the historical data on which Oberlin's analysis was based included histology (alveolar/non alveolar) but not *PAX::FOXO1* gene fusion status. More recently, it was shown that fusion status and Oberlin score were highly correlated [4, 5]. A recent study from the Children's Oncology Group (COG) using survival tree regression for EFS found that among RMS patients, *FOXO1* fusion status was the most important prognostic characteristic in M1 RMS after metastatic status [6].

Whereas clinical prognostic factors have been defined following first relapse of localised RMS [7, 8, 9], less is known about factors predicting survival following first relapse or progression of M1 RMS, although histologic subtype, disease group and disease stage influenced outcomes in a COG study of relapse of localised and M1 RMS [2]. The aim of this study was to use pooled data from the International Soft Tissue saRcoma ConsorTium (INSTRuCT), a soft tissue sarcoma data commons that includes US and European data from children and young people with RMS [10], to evaluate OS in M1 RMS following first event, describe the clinical features associated with survival after relapse/progression and, if possible, to identify prognostic indicators or develop a prognostic tool to aid decision making for this cohort.

## 2 Materials and Methods

The Upfront Cohort (UC) included patients with RMS entered in the INSTRuCT database who fulfilled the following eligibility criteria: histologically proven, paediatric type (i.e., excluding pleomorphic), rhabdomyosarcoma at first diagnosis; one or more distant metastatic organ sites at first presentation (M1 disease); aged from birth to 40 years at initial diagnosis. The cohort of interest (First Event Cohort; FEC) included patients within the study population with a documented event of relapse (local/locoregional, metastatic or combined relapse) or disease progression. Patients with other event types (which included death and second malignancy) and those with follow up of < 1 day from the event were excluded. Included patients were diagnosed between 1991 and 2016. Data were transferred from INSTRuCT on 01/09/2022.

Patients with M1 RMS were included from the following co‐operative group studies: COG trials ARST08P1 [11], D9802 [12], ARST 0431 [13] and D9803 [14]; EpSSG trial MTS 2008 [3]; CWS studies CWS 91 addendum (treatment as per MMT4‐99 and MMT4‐91) [15], CWS‐96 HD [16], CWS‐DOKIV and CWS‐IV‐2002 [17]; International Society of Paediatric Oncology (SIOP) MMT 98 trial [18]; Italian Cancer Group (ICG) RMS 4.99 trial [19].

INSTRuCT data (version 1.0) included information on contributor group and which clinical trial the patient was enrolled on (see Table S1). It also included patient and tumour characteristics. Chemotherapy treatment or treatment options could be inferred from knowledge of the clinical trials/trial arm included, but there were no individual patient data on systemic therapy received, secondary surgery or radiotherapy. Except for type of event and extent of relapse/progression, no data on clinical characteristics at relapse/progression or the treatment administered after the first event were available. In the INSTRuCT data dictionary (see https://commons.cri.uchicago.edu/instruct/) relapse is defined as “the return of a disease after a period of remission” and progression is defined as “a process that manifests as the worsening of a disease over time”.

The clinical features of UC and FEC were described as absolute numbers and percentages and compared using Chi‐squared or Fisher exact test according to the frequency distribution. Patients alive with > 3 years of follow up from the first event of relapse/progression were considered “disease free”. The baseline characteristics, type of relapse and time to relapse of these “disease free” patients were compared with those who died in the same way. Survival was calculated using the Kaplan–Meier method. For the UC, EFS was defined as the time from diagnosis [3, 15, 16] or study entry [11, 12, 13] or treatment initiation [14, 17] to first event of death, relapse/progression or second malignancy and OS was defined as the time from diagnosis [3, 15, 16] or study entry [11, 12, 13] or treatment initiation [14, 17] to death or end of follow up. Univariate and multivariable analyses were conducted in FEC to investigate the impact of each factor on OS. The time was calculated from first relapse/progression. Three‐year OS values were reported together with the 95% confidence interval (CI). A multivariable Cox regression model stratified by time of relapse, which did not respect the proportional hazard assumption, was estimated, and for the variables with a significant impact after stepwise selection, the Hazard Ratio (HR) and relative 95% confidence limits (CL) were reported. The analyses were performed with the statistical software SAS version 9.4 (SAS Institute Inc., Cary, NC, USA).

## 3 Results

**FIGURE 1:**
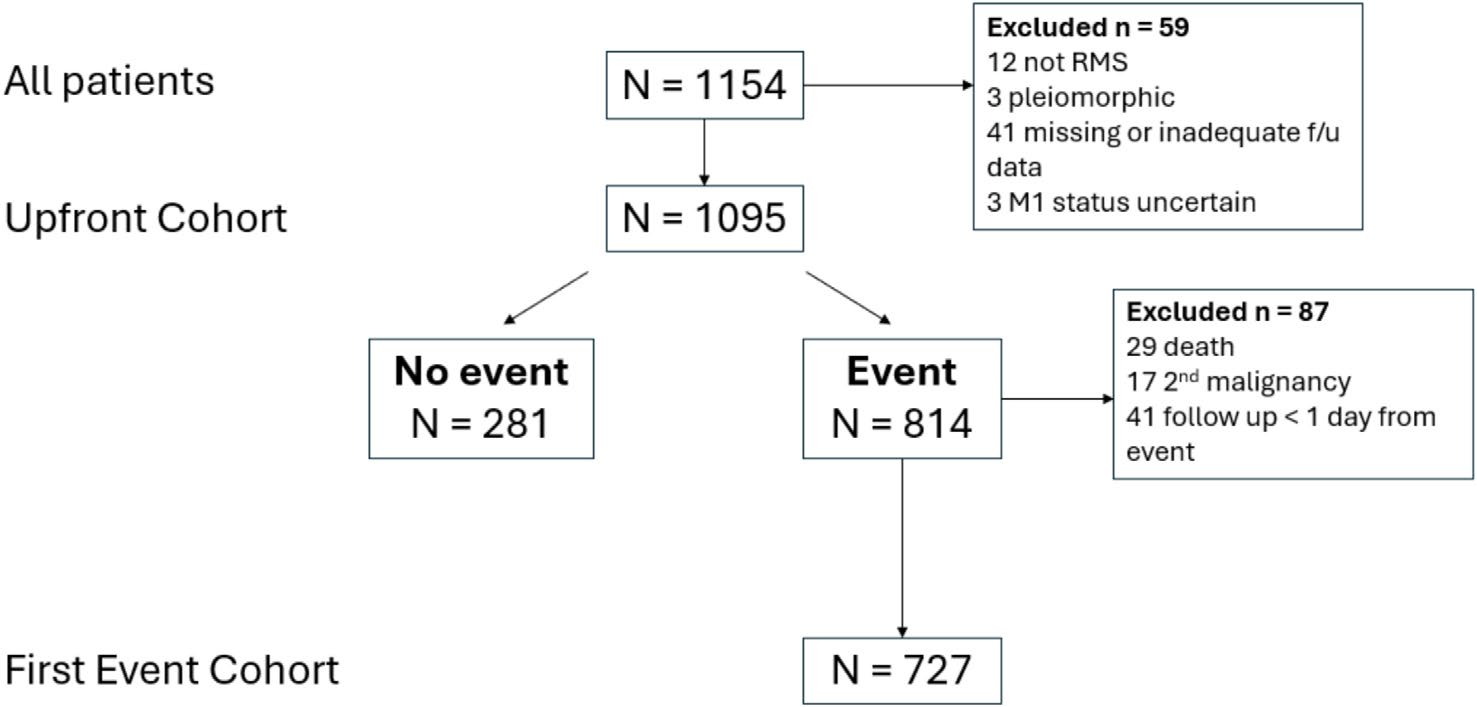
CONSORT diagram showing selection of Upfront and First Event Cohorts from INSTRuCT database.

At the time of data transfer, the INSTRuCT database included 6969 patients with RMS. Of these, 1154 patients (16.5%) had M1 disease and 1095 (15.7%) fulfilled the eligibility criteria for the study population (UC; Figure 1). The contribution to UC by co‐operative group is shown in Table S1. Eight hundred and fourteen patients had an event, of whom 727 had a first event of relapse/progression and were eligible for inclusion in FEC (Figure 1). Eighty‐seven patients with an event were excluded for the following reasons: 29 first event was death, 17 second malignancy, 41 follow up < 1 day from event.

The clinical features of UC are shown in Table 1. The median age at first presentation was 11.0 years (range 5 days‐30.4 years, interquartile range 5.0–15.8 years) and 57.8% had alveolar histology. Of note, less than 50% of patients had known *PAX::FOXO1* gene fusion status. The most common disease sites were “other” (32.7%), extremities (25.8%) and parameningeal (17.0%), 75.1% of primary tumours were > 5 cm in maximal dimension, and 56.9% had locoregional nodal involvement (clinical and/or pathological nodal involvement, N1). The most frequent presentation was with a single metastatic organ site (45.4%) but a maximum of 7 metastatic sites were documented (in 2 patients). Bone was the most common metastatic site (42.0%), followed closely by lung (40.2%; Table 2). Bone marrow, lymph node, soft tissue, pleura and other metastatic sites were seen in 32.8%, 32.1%,10.9% and 25% of patients respectively, with 0.9% unknown (Table 2). The most frequent single metastatic site was lung (17% of patients). The distribution of Oberlin factors was: 9.4%, 27.3%, 28.5%, 22.3% and 11.6%, for 0–4 factors respectively (0.9% unknown). The number of documented metastatic sites did not vary significantly by year of diagnosis but there was a trend towards more sites being documented from the year 2000 onwards (data not shown).

**TABLE 1:** Baseline clinical characteristics of Upfront Cohort and First Event Cohort.

	Upfront Cohort (1095)	First Event Cohort (727)	*p*
	*N* (%)	*N* (%)	
Sex			
Female	505 (46.1)	337 (46.4)	0.9211
Male	590 (53.9)	390 (53.6)	
Year of diagnosis			
1991–1994	31 (2.8)	23 (3.2)	0.7480
1995–1999	193 (17.6)	114 (15.7)	
2000–2004	249 (22.7)	175 (24.1)	
2005–2009	227 (20.7)	143 (19.7)	
2010–2016	395 (36.1)	272 (37.4)	
Age at diagnosis			
< 1 year	25 (2.3)	17 (2.3)	0.0082
1–9 years	479 (43.7)	260 (35.8)	
10–20 years	572 (52.2)	434 (59.7)	
≥ 21 years	19 (1.7)	16 (2.2)	
Histology			
Favourable RMS	424 (38.7)	204 (28.1)	< 0.0001
Unfavourable RMS^a^	671 (61.3)	523 (71.9)	
PAX‐FOXO1 fusion status			
Positive	335 (30.7)	278 (38.2)	0.0011^b^
*FOXO1‐PAX3*	*249 (74.1)*	*210 (75.5)*	
*FOXO1‐PAX7*	*37 (11.0)*	*26 (9.4)*	
*FOXO1*	*49 (14.6)*	*42 (15.1)*	
Negative	182 (16.6)	91 (12.5)	
Unknown	578 (52.8)	358 (49.2)	
Tumour site^d^			
Favourable	402 (36.7)	226 (31.1)	0.0134
Unfavourable	693 (63.3)	501 (68.9)	
Tumour size			
≤ 5 cm	220 (20.1)	157 (21.6)	0.4180*
> 5 cm	822 (75.1)	533 (73.3)	
Unknown	53 (4.8)	37 (5.1)	
T‐invasiveness			
T1	161 (14.7)	93 (12.8)	0.2402*
T2	903 (82.5)	615 (84.6)	
Tx	31 (2.8)	19 (2.6)	
Local/locoregional N involvement^e^			
N0	388 (35.4)	237 (32.6)	0.2380*
N1	623 (56.9)	430 (59.2)	
Nx	84 (7.7)	60 (8.2)	
Number of metastatic sites			
1 site	497 (45.4)	268 (36.9)	0.0240*
2 sites	283 (25.8)	212 (29.2)	
3 sites	170 (15.5)	133 (18.3)	
4 sites	75 (6.8)	63 (8.7)	
5 sites	45 (4.1)	35 (4.8)	
6 sites	13 (1.2)	12 (1.7)	
7 sites	2 (0.2)	2 (0.3)	
Unknown^c^	10 (0.9)	2 (0.3)	
Oberlin score			
0 factors	103 (9.4)	44 (6.1)	< 0.0001*
1 factor	299 (27.3)	153 (21.1)	
2 factors	312 (28.5)	210 (28.9)	
3 factors	244 (22.3)	202 (27.8)	
4 factors	127 (11.6)	116 (16.0)	
Unknown	10 (0.9)	2 (0.3)	

*Note:* Chi‐square test used for all comparisions.

^a^ Alveolar 633, mixed embryonal/alveolar 4, not classified 34.

^b^ Comparison of PAX‐FOX01 fusion positive versus negative, and excluding patients with unknown fusion status.

^c^ Patients with a specific primary tumour site and no details on metastatic site.

^d^ Favourable site: orbit, all head and neck, all genitourinary. Unfavourable site: extremity and “other”.

^e^ Loco‐regional nodal involvement (N1) included patients with clinical and/or pathological evidence of lymph node involvement. Nx; lymph node status unknown.

* Patients with unknown tumour size, T‐status, nodal status, metastatic site and Oberlin score are excluded from the distribution tests.

**TABLE 2:** Metastatic sites in Upfront Cohort.

	*N* (%)
Bone	460 (42.0)
Alone	53
With other metastatic sites	407
Bone marrow	359 (32.8)
Alone	72
With other metastatic sites	287
Bone and bone marrow	239 (21.8)
Alone	64
With other metastatic sites	175
Lung	440 (40.2)
Alone	187
With other metastatic sites	253
Lymph nodes	352 (32.1)
Alone	83
With other metastatic sites	269
Pleural effusion	119 (10.9)
Alone	15
With other metastatic sites	104
Soft tissue	186 (17.0)
Alone	19
With other metastatic sites	167
Other^a^	274 (25.0)
Alone	68
With other metastatic sites	206
Unknown	10 (0.9)
Alone	10^b^

^a^ 9 with brain metastasis, 2 with meningeal metastasis, 5 with peritoneal metastasis. For the remaining, specification is missing.

^b^ 10 patients with a specific primary tumour site and no details on metastatic site.

Among 631 patients with documented primary surgery (ranging from biopsy only to complete resection), 15 (1.4%) had R0 resection (clear margins), 21 (1.9%) had R1 resection (microscopic residual disease) and 595 (94.3%) had R2 resection (residual macroscopic disease). Data on primary surgery were missing on the remaining 464 patients (42.4%). There were no data on secondary surgery. The majority of chemotherapy regimens used in the contributor trials were alkylating agent‐based (ifosfamide/cyclophosphamide) and some trials included maintenance chemotherapy.

**FIGURE 2:**
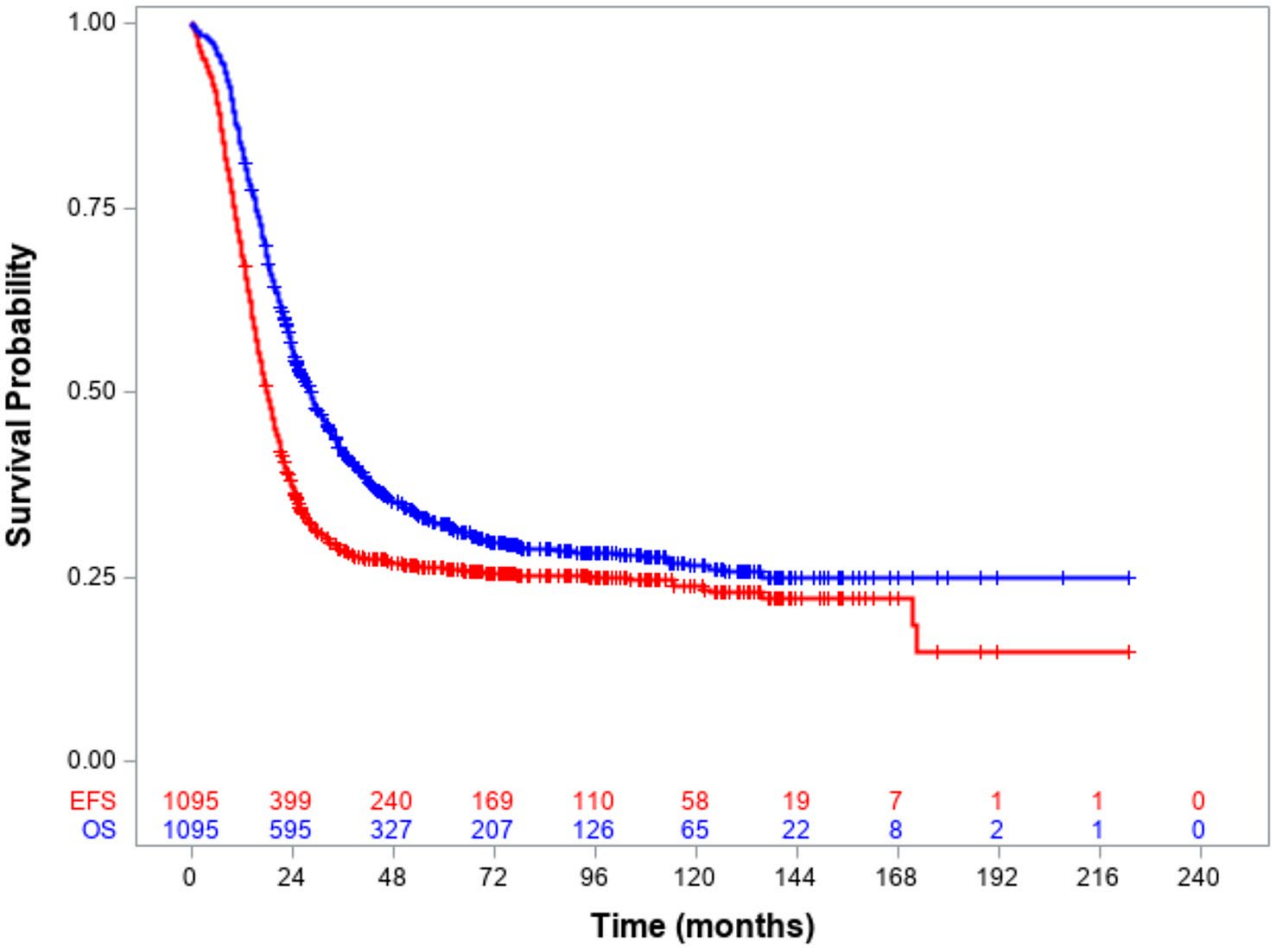
Event free survival and overall survival from diagnosis, Upfront Cohort. 5‐year OS for the whole Upfront Cohort of metastatic patients was 32.0% (95% CI 29.2–34.9), 5‐year EFS considering all events was 27.5% (95% CI 24.8–30.2).

Median follow up from diagnosis for alive patients was 6.4 years (range 1.1–18.6 years). The 5‐year OS from diagnosis of UC was 32.0% (95% CI 29.2–34.9) and 5‐ year EFS from diagnosis was 27.5% (95% CI 24.8–30.2) (Figure 2). The median age at first event for the UC cohort was 13.7 years (range 0.1–34.9 years) and median time to event was 13.9 months (range 0.03–172.6 months). Over half of all events (414/814, 50.9%) involved relapse at one or more metastatic sites. Isolated local or regional relapse occurred in 100 (12.3%) and 22 (2.7%) events respectively and 232 events (28.5%) were reported as progressive disease.

Clinical characteristics of the 727 patients included in FEC are shown in Table 1. Compared to UC patients, FEC patients were significantly older (61.7% vs. 53.9% > 10 years, *p* = 0.0082) with higher proportions of unfavourable histology (*p* < 0.0001), fusion positive disease (*p* = 0.0010), unfavourable primary tumour site (*p* = 0.0134), more metastatic sites (*p* = 0.024) and a higher frequency of Oberlin scores of 3–4 (*p* < 0.0001).

**FIGURE 3:**
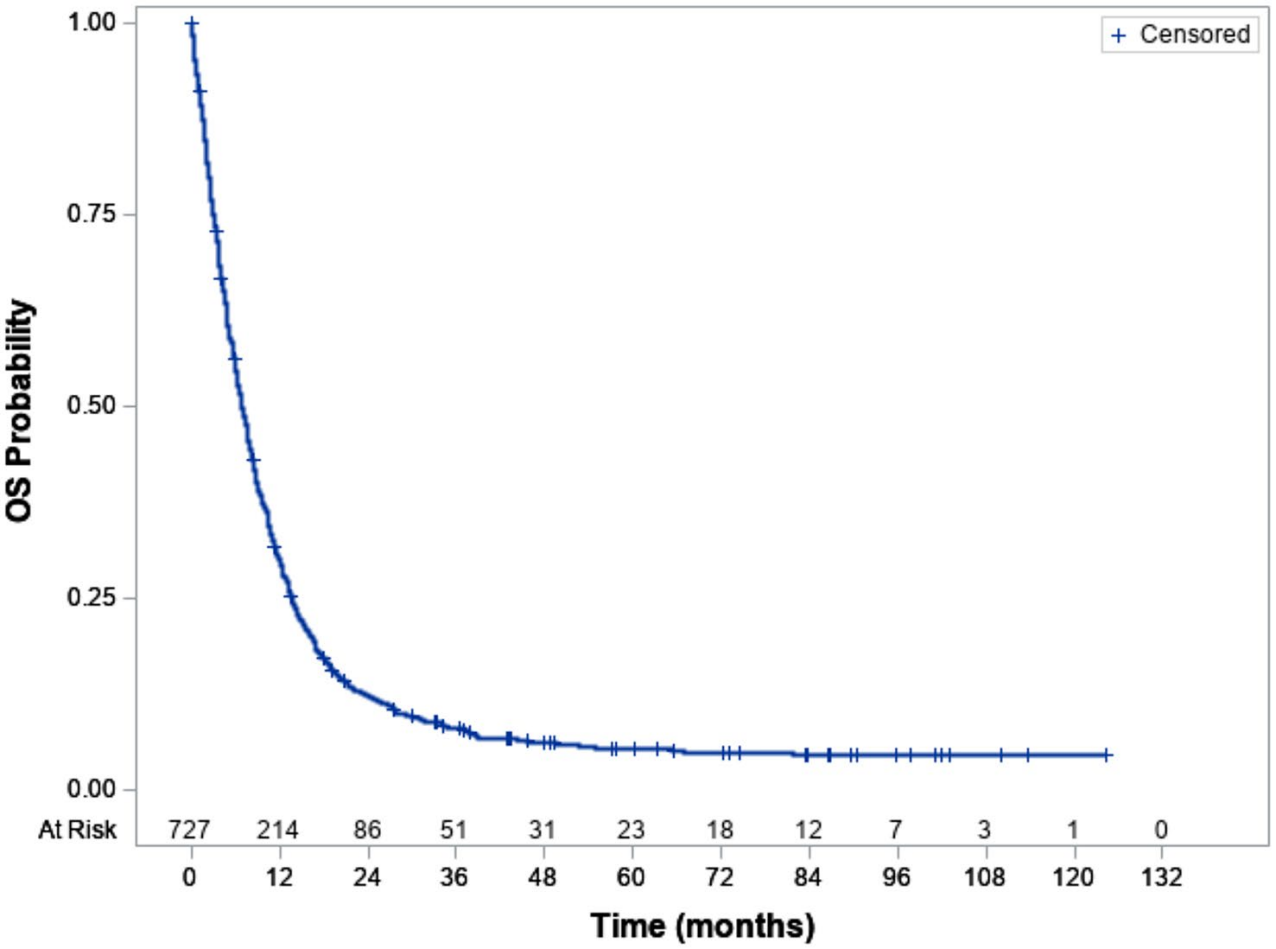
Overall Survival from first event, First Event Cohort. 3‐year OS for the First Event Cohort with disease progression or relapse was 8.0 (95% CI 6.1–10.2).

Overall survival for FEC from disease relapse/progression to last follow‐up is shown in Figure 3. Three‐year OS from the first event was only 8.0% (95% CI 6.1–10.2). Since the survival curve for FEC flattened from 3 years (Figure 3), we considered patients alive > 3 years from first event as “disease free”. Fifty of 727 patients were alive at last follow up: 34 FEC patients (4.7% of FEC) were “disease free” and 16 patients were alive with 3 years or less follow up (range 0.03–34.4 months). Deaths in FEC were due to disease progression (652; 96.3%), treatment related mortality (4; 0.6%), second malignancy (1; 0.1%), other cause (18; 2.7%) or unknown reason (2; 0.3%).

FEC patients had the same distribution by cooperative group as UC (Table S2). The clinical characteristics and metastatic sites at first presentation of “disease free” versus deceased FEC patients were compared to investigate whether any features of “disease free” patients after first relapse/progression could be identified (Table 3). “Disease free” patients were significantly more likely at first diagnosis to be younger (*p* = 0.0031), have negative locoregional lymph nodes (N0; *p* = 0.0013), have fewer metastatic sites (*p* = 0.0006) and lower Oberlin scores (*p* < 0.0001). They were also significantly less likely to have bone (*p* < 0.0001), bone marrow (*p* = 0.0082) or combined bone/bone marrow (*p* = 0.0003) disease at diagnosis. In addition, “disease free” patients were more likely to have had the first event > 18 months from diagnosis (*p* < 0.0001) but the type of event they had (local, locoregional or metastatic) was not significant.

**TABLE 3:** Baseline clinical characteristics of deceased patients and alive pts. with > 3 years of follow up from First Event Cohort.

	Alive > 3 years	Deceased	*N* = 711^a^	*p*
	*N* = 34	*N* = 677		
	*N* (%)	*N* (%)	Total (%)	
Sex				
Female	13 (38.2)	316 (46.7)	329 (46.3)	0.3354
Male	21 (61.8)	361 (53.3)	382 (53.7)	
Age at diagnosis				
< 1 year	3 (8.8)	12 (1.8)	15 (2.1)	*0.0031*
1–9 years	19 (55.9)	237 (35.0)	256 (36.0)	
10–20 years	12 (35.3)	412 (60.9)	424 (59.6)	
≥ 21 years	—	16 (2.4)	16 (2.3)	
Histology				
Favourable RMS	13 (38.2)	188 (27.8)	201 (28.3)	0.1860
Unfavourable RMS	21 (61.8)	489 (72.2)	510 (71.7)	
Fusion status				
FOXO1 positive	11 (32.3)	262 (38.7)	273 (40.3)	0.1412^c^, *
*FOXO1‐PAX3*	*5 (45.5)*	*203 (77.5)*	*208 (76.2)*	
*FOXO1‐PAX7*	*2 (18.2)*	*22 (8.4)*	*24 (8.8)*	
*FOXO1*	*4 (36.4)*	*37 (14.1)*	*41 (15.0)*	
FOXO1 negative	7 (20.6)	81 (12.0)	88 (12.4)	
Unknown	16 (47.1)	334 (49.3)	350 (49.2)	
Tumour site				
Favourable	12 (35.3)	210 (31.0)	222 (31.2)	0.5997
Unfavourable	22 (64.7)	467 (69.0)	489 (68.8)	
Tumour size				
≤ 5 cm	10 (29.4)	143 (21.1)	153 (21.5)	0.1916*
> 5 cm	21 (61.8)	501 (74.0)	522 (73.4)	
Unknown	3 (8.8)	33 (4.9)	36 (5.1)	
T‐invasiveness				
T1	8 (23.5)	80 (11.8)	88 (12.4)	0.0518*
T2	26 (76.5)	579 (85.5)	605 (85.1)	
Tx	—	18 (2.7)	18 (2.5)	
Local/locoregional N involvement^b^				
N0	21 (61.8)	211 (31.2)	232 (32.6)	0.0013*
N1	11 (32.4)	409 (60.4)	420 (59.1)	
Nx	2 (5.9)	57 (8.4)	59 (8.3)	
Number of metastatic sites				
1 site	24 (70.6)	235 (34.7)	259 (36.4)	*0.0006* *
2 sites	6 (17.7)	203 (30.0)	209 (29.4)	
3 sites	—	130 (19.2)	130 (18.3)	
4 sites	2 (5.9)	61 (9.0)	63 (8.9)	
5 sites	—	34 (5.0)	34 (4.8)	
6 sites	—	12 (1.8)	12 (1.7)	
7 sites	—	2 (0.3)	2 (0.3)	
Unknown	2 (5.9)	—	2 (0.3)	
Type of metastatic site				
Bone	4 (11.8)	355 (52.4)	359 (50.5)	< *0.0001* *
Bone marrow	6 (17.7)	273 (40.3)	279 (39.2)	0.0082
Bone + bone marrow	1 (2.9)	193 (28.5)	194 (27.3)	*0.0003*
Lung	12 (35.3)	252 (37.2)	264 (37.1)	0.8203
Lymph nodes	7 (20.6)	241 (35.6)	248 (34.9)	0.0731
Pleural effusion	4 (11.8)	89 (13.2)	93 (13.1)	*1.0000*
Soft tissue	5 (14.7)	131 (19.4)	136 (19.1)	*0.6562*
Other	6 (17.7)	190 (28.1)	196 (27.6)	0.1847
Unknown	2 (5.9)	—	2 (0.3)	—
Oberlin score				
0 factors	6 (17.7)	37 (5.5)	43 (6.1)	< *0.0001* *
1 factor	14 (41.2)	135 (19.9)	149 (21.0)	
2 factors	8 (23.5)	197 (29.1)	205 (28.8)	
3 factors	2 (5.9)	196 (29.0)	198 (27.9)	
4 factors	2 (5.9)	112 (16.5)	114 (16.0)	
Unknown	2 (5.9)	—	2 (0.3)	
Oberlin score				
0–2 factors	28 (82.4)	369 (54.5)	397 (55.8)	*0.0012*
3‐4 factors	6 (17.6)	308 (45.5)	314 (44.2)	
Type of first event				
Local relapse	9 (26.5)	89 (13.2)	98 (13.8)	0.0927
Loco‐regional relapse	1 (2.9)	19 (28.7)	20 (2.8)	
Metastatic relapse	19 (55.9)	382 (56.4)	401 (56.4)	
Progressive disease	5 (14.7)	187 (27.6)	192 (27.0)	
Time to first event				
≤ 18 months	12 (35.3)	481 (71.1)	493 (69.3)	< 0.0001
> 18 months	22 (64.7)	196 (28.9)	218 (30.7)	

*Note:* Chi‐square test; *Fisher exact test.*

^a^ 16 patients alive with < 3 years follow up were excluded from this comparison: see Results section.

^b^ Local/locoregional nodal involvement (N1) included patients with clinical and/or pathological evidence of lymph node involvement. Nx; lymph node status unknown.

^c^ Comparison of PAX‐FOXO1 fusion positive versus negative, and excluding patients with unknown fusion status.

* Patients with unknown fusion status, tumour size, T‐status, nodal status, metastatic site and Oberlin score are excluded from the distribution tests.

To further examine the factors most significant for 3‐year OS from first event, univariate analysis was performed on 637 patients in FEC following exclusion of 90 patients with unknown values for one or more of nodal involvement (60), T‐status (19), tumour size (37) or number of metastatic sites (2). All FEC patients were included in this analysis whether or not they had 3 years follow up from first event. Factors with *p* < 0.25 at univariate analysis were: age at diagnosis (< 1 or ≥ 10 years vs. 1–9 years), locoregional nodal involvement, tumour invasiveness, number of metastatic sites (1–2 vs. ≥ 3 sites), Oberlin score (0–1 factors vs. ≥ 2), type of first event (local/locoregional vs. metastatic vs. progression) and time to first event (≤ 18 months vs. > 18 months; Table S3). These factors were then included in multivariable analysis, excluding age and number of metastatic sites owing to their relationship to Oberlin risk factors. After stepwise selection, the two factors significant for poorer OS were Oberlin score ≥ 2 (HR 1.295, 95% CL 1.07–1.57, *p* = 0.0074) and involvement of loco‐regional nodes (HR 1.28, 95% CL 1.08–1.52, *p* = 0.0053). The baseline risk function was different according to the time to first event: ≤ 18 months and > 18 months.

## 4 Discussion

The 5‐year EFS of 27.5% and OS of 32.0% in this largest cohort of M1 RMS reported to date are consistent with the previously reported pooled analysis of 788 patients, where 3‐year EFS and OS were 27% and 34% respectively (Oberlin 2008). There was some (< 20%) overlap between the Oberlin and INSTRuCT cohorts which included 46 and 69 patients respectively from the RMS4.99 study and 127 and 106 patients respectively from the MMT 98 study. Survival curves in the current study (Figure 2) and in Oberlin's pooled analysis [1] suggest that almost all first events occurred within 3 years from diagnosis. This INSTRuCT analysis includes more recent cohorts with slightly better outcomes from EpSSG studies (3‐year EFS and OS of 34.9% (95% CI, 29.1 to 40.8) and 47.9% (95% CI, 41.6 to 53.9) respectively) [3] and the COG ARST0431 study (3‐year EFS and OS of 38% (95% CI, 29 to 48%) and 56% (95% CI, 46 to 66%) respectively) [13] Nevertheless, outcomes in these patients overall remain poor. About half of the events in UC were reported to include relapse at one or more metastatic organ sites, contributing to the difficulty of salvage. The proportion of patients with progressive disease appeared high at 28.5%, but a limitation on the interpretation of these data is that patients were reported as having relapse, progression or both affecting primary and/or nodal and/or metastatic sites making it difficult to separate relapse and progression cleanly. For patients with multiple disease sites this heterogeneity in event types is not unexpected.

No study has previously focussed exclusively on predictors of outcome following first progression or relapse of metastatic RMS. The finding that relapsed/refractory (FEC) patients were significantly older with significantly more unfavourable histology, fusion positive disease, unfavourable site, number of metastatic sites and Oberlin scores of 3–4 (Table 1) confirmed previously described findings [1, 6]. Of these, age, unfavourable site and number of metastatic sites also contribute to Oberlin scores. However, several authors have demonstrated the importance of *PAX::FOXO1* fusion status in M1 RMS [4, 5, 6] and unfavourable histology links closely to fusion positive RMS. Almost half of UC patients had unknown fusion status reflecting the relatively recent practice of fusion status testing; for some time, fusion status was only routinely performed for tumours with alveolar (unfavourable) histology. Currently it is recommended that all patients should undergo fusion status testing and in future analyses fusion status may become significant in a revision of Oberlin risk factors.

A key finding in the current study is the demonstration that 3‐year OS of FEC patients was only 8%, consistent with the clinical impression of dismal outcomes in these patients. This is similar to the 3‐year OS of 10% reported by the CWS group in 129 patients with relapse of metastatic RMS [4] and Pappo's report of 12% 5‐year OS in relapsed metastatic embryonal RMS and 3% in patients with group 2, 3 and 4 alveolar RMS [2]. A recent study from 5 COG institutions has shown that intensity of alkylator treatment impacted on outcome in patients with relapsed RMS who had low risk but not high risk (mainly metastatic) RMS [20].

Almost all deaths in FEC (96.3%) were disease related. The OS curve following first event for FEC (Figure 3) demonstrated that almost all deaths occurred with within 3 years. For this reason, we considered patients alive > 3 years from first event as “disease free” and sought to examine whether adverse prognostic factors for outcome following first event can be determined in metastatic RMS, similar to studies in first relapse of localised RMS where risk factors for survival after relapse could be identified from clinical factors at first diagnosis, type of chemotherapy treatment, clinical characteristics at relapse and time to relapse [7, 8, 9].

Patients who were “disease free” were significantly more likely to have a first event > 18 months from diagnosis, similar to previous reports in localised RMS that patients with later events have better outcomes [9, 21]. They had fewer Oberlin risk factors at first presentation. They were more likely to have negative locoregional lymph nodes which was previously identified as significant in univariable but not multivariable analysis [1]. In our Cox regression model stratified for time to relapse, we found that locoregional nodal involvement at diagnosis was associated with significantly worse outcome along with > 2 Oberlin factors at diagnosis. These data seem to suggest that the few patients who survived first progression/relapse were more likely to have characteristics that would indicate lower risk of relapse initially, although the lymph node data are intriguing, particularly as we did not find a significant difference in the incidence of N0 between UC and FEC (Table 1, *p* = 0.2380).

There were several major limitations of our study. We had no data on radiotherapy treatment, details of chemotherapy nor surgery beyond initial surgery in first line treatment. Except for extent of relapse/progression, we had no data at all on clinical characteristics at relapse nor details of systemic or local treatment after relapse. We were therefore unable to determine whether frontline treatment and treatment at progression/relapse impacted on outcome. Additionally biological data were limited to fusion status in a subset of patients. Moreover, the finding of some very early events (range 0.03–172.6 months) was unexpected and may have reflected the definition of time to event (from diagnosis, or study entry, or treatment initiation), for example if there was delay between diagnosis and study entry or start of treatment. However, the documented poor outcomes do support the international RMS community's commitment to continued consideration of such patients for inclusion in early phase clinical trials wherever possible.

Randomised studies in relapsed RMS from the COG group [22, 23] have excluded favourable risk patients (botryoid histology at initial diagnosis or Stage 1 or Clinical Group I embryonal RMS at initial diagnosis, not treated with cyclophosphamide, and who recurred either locally or regionally) but have not stratified the randomisation by clinical risk factors. However, in the COG ARST0921 study a Cox proportional hazards regression model was used to calculate the hazard ratio for treatment failure after adjusting for histology [23].

Defachelles [24] reported a randomised study in refractory/relapsed rhabdomyosarcoma from the EpSSG group where patients were stratified from the outset for refractory versus relapsed disease and country of treatment with stratification by previous radiotherapy and metastases at study entry (yes/no) added later. Survival analyses were adjusted for histological subtype (alveolar vs. non alveolar) and type of relapse (metastatic vs. locoregional) and disease status (relapsed vs. refractory disease). This study did not find any treatment effect on objective response, progression free survival or OS by disease status, metastatic vs. locoregional relapse or alveolar vs. non alveolar histology but did not differentiate localised vs. metastatic disease at frontline presentation. In the current EpSSG study in relapsed RMS [25], patient stratification includes prior radiotherapy (yes/no), relapse type (metastatic/locoregional), *PAX::FOXO 1* fusion status (positive or negative) and age at diagnosis (≤ 10, > 10 and ≤ 18, > 18). There is clearly an urgent need for clinical trials in relapsed RMS to have standardised stratification factors which, in addition to other clinical and biological factors, may also need to include localised versus metastatic disease at first presentation and potentially metastatic risk group by Oberlin risk factors.

In conclusion, outcomes following first progression/relapse of metastatic RMS are dismal and such patients should be considered for early phase clinical trials wherever possible. While we have been unable to identify prognostic factors to guide clinical decision making and conversations with patients and families in progression/relapse of M1 RMS, “disease free” patients were enriched for better prognostic factors at first diagnosis and were more likely to relapse > 18 months from diagnosis. This study highlights the need for internationally agreed stratification factors for clinical trials in relapsed RMS.

## Author Contributions

**Ajla T. Wasti:** conceptualization (equal), data curation (equal), project administration (lead), writing – original draft (equal), writing – review and editing (equal). **Gianni Bisogno:** data curation (equal), formal analysis (equal), methodology (equal), project administration (supporting), writing – review and editing (equal). **Beatrice Coppadoro:** data curation (equal), formal analysis (lead), methodology (lead), writing – original draft (equal), writing – review and editing (equal). **Ilaria Zanetti:** data curation (equal), formal analysis (lead), methodology (lead), writing – original draft (equal), writing – review and editing (equal). **Martin Ebinger:** data curation (equal), writing – original draft (equal), writing – review and editing (equal). **Amadeus T. Heinz:** data curation (equal), writing – review and editing (equal). **Henry C. Mandeville:** conceptualization (supporting), supervision (supporting), writing – review and editing (equal). **Rita Alaggio:** data curation (equal), writing – review and editing (equal). **Michela Casanova:** data curation (equal), writing – review and editing (equal). **Sheila E. J. Terwisscha van Scheltinga:** data curation (equal), writing – review and editing (equal). **Rick R. van Rijn:** data curation (equal), writing – review and editing (equal). **Veronique Minard‐Colin:** data curation (equal), writing – review and editing (equal). **Daniel Orbach:** data curation (equal), writing – review and editing (equal). **Natalie B. Collins:** data curation (equal), writing – review and editing (equal). **Wei Xue:** data curation (equal), validation (equal), writing – review and editing (equal). **Rajkumar Venkatramani:** data curation (equal), writing – original draft (equal), writing – review and editing (equal). **Johannes H. M. Merks:** conceptualization (equal), data curation (equal), formal analysis (supporting), methodology (supporting), writing – original draft (equal), writing – review and editing (equal). **Julia C. Chisholm:** conceptualization (lead), data curation (equal), formal analysis (supporting), methodology (supporting), project administration (equal), supervision (lead), writing – original draft (lead), writing – review and editing (equal).

## Funding

AW and JCC were supported by The Royal Marsden Cancer Charity through The Giant Pledge. This work represents independent research supported by the National Institute for Health Research (NIHR) Biomedical Research Centre. The views expressed are those of the authors and not necessarily those of the NIHR or the Department of Health and Social Care. ME and AH were supported by the German Children's Cancer Foundation (DKS).

The EpSSG Data Centre is supported in part by Alice's Arc.

## Conflicts of Interest

The authors declare no conflicts of interest.

## Supporting information


**Table S1.** Upfront Cohort: Studies list by data contributor.
**Table S2.** Studies list by data contributor in First Event Cohort.
**Table S3.** Univariate analysis of Overall Survival from first event in First Event Cohort (*n* = 637*).

## Data Availability

Data available on request from the authors.

## References

[1] O. Oberlin , A. Rey , E. Lyden , et al., “Prognostic Factors in Metastatic Rhabdomyosarcomas: Results of a Pooled Analysis From United States and European Cooperative Groups,” J Clin Oncol 26 (2008): 2384–2389. 18467730 10.1200/JCO.2007.14.7207 PMC4558625

[2] A. S. Pappo , J. R. Anderson , W. M. Crist , et al., “Survival After Relapse in Children and Adolescents With Rhabdomyosarcoma: A Report From the Intergroup Rhabdomyosarcoma Study Group,” J Clin Oncol 17, no. 11 (1999): 3487–3493, 10.1200/JCO.1999.17.11.3487. 10550146

[3] R. A. Schoot , J. C. Chisholm , M. Casanova , et al., “Metastatic Rhabdomyosarcoma: Results of the European *Paediatric* Soft Tissue Sarcoma Study Group MTS 2008 Study and Pooled Analysis With the Concurrent BERNIE Study,” J Clin Oncol 10, no. 32 (2022): 3730–3740, 10.1200/JCO.21.02981. PMC9649279 35709412

[4] A. T. Heinz , A. Schönstein , M. Ebinger , et al., “Significance of Fusion Status, Oberlin Risk Factors, Local and Maintenance Treatment in Pediatric and Adolescent Patients With Metastatic Rhabdomyosarcoma: Data of the European Soft Tissue Sarcoma Registry SoTiSaR,” Pediatr Blood Cancer 71, no. 1 (2024): e30707, 10.1002/pbc.30707. 37814424

[5] E. R. Rudzinski , J. R. Anderson , Y. Y. Chi , et al., “Histology, Fusion Status, and Outcome in Metastatic Rhabdomyosarcoma: A Report From the Children's Oncology Group,” Pediatr Blood Cancer 64, no. 12 (2017): e26645, 10.1002/pbc.26645. PMC5647228 28521080

[6] E. Hibbitts , Y. Y. Chi , D. S. Hawkins , et al., “Refinement of Risk Stratification for Childhood Rhabdomyosarcoma Using FOXO1 Fusion Status in Addition to Established Clinical Outcome Predictors: A Report From the Children's Oncology Group,” Cancer Med 8, no. 14 (2019): 6437–6448, 10.1002/cam4.2504. 31456361 PMC6797586

[7] S. Mazzoleni , G. Bisogno , A. Garaventa , et al., “Outcomes and Prognostic Factors After Recurrence in Children and Adolescents With Nonmetastatic Rhabdomyosarcoma,” Cancer 104, no. 1 (2005): 183–190, 10.1002/cncr.21138. 15895378

[8] T. M. Dantonello , C. Int‐Veen , P. Winkler , et al., “Initial Patient Characteristics Can Predict Pattern and Risk of Relapse in Localized Rhabdomyosarcoma,” J Clin Oncol 20, no. 3 (2008): 406–413, 10.1200/JCO.2007.12.2382. 18202417

[9] J. C. Chisholm , J. Marandet , A. Rey , et al., “Prognostic Factors After Relapse in Nonmetastatic Rhabdomyosarcoma: A Nomogram to Better Define Patients Who Can Be Salvaged With Further Therapy,” J Clin Oncol 1, no. 10 (2011): 1319–1325, 10.1200/JCO.2010.32.1984. 21357778

[10] K. D. Wyatt , S. Birz , D. S. Hawkins , et al., “Creating a Data Commons: The INternational Soft Tissue SaRcoma ConsorTium (INSTRuCT),” Pediatr Blood Cancer 69, no. 11 (2022): e29924, 10.1002/pbc.29924. 35969120 PMC9560864

[11] S. Malempati , B. J. Weigel , Y. Y. Chi , et al., “The Addition of Cixutumumab or Temozolomide to Intensive Multiagent Chemotherapy Is Feasible but Does Not Improve Outcome for Patients With Metastatic Rhabdomyosarcoma: A Report From the Children's Oncology Group,” Cancer 15, no. 2 (2019): 290–297, 10.1002/cncr.31770. PMC6329653 30351457

[12] A. S. Pappo , E. Lyden , P. Breitfeld , et al., “Two Consecutive Phase II Window Trials of Irinotecan Alone or in Combination With Vincristine for the Treatment of Metastatic Rhabdomyosarcoma: The Children's Oncology Group,” J Clin Oncol 1, no. 4 (2007): 362–369, 10.1200/JCO.2006.07.1720. 17264331

[13] B. J. Weigel , E. Lyden , J. R. Anderson , et al., “Intensive Multiagent Therapy, Including Dose‐Compressed Cycles of Ifosfamide/Etoposide and Vincristine/Doxorubicin/Cyclophosphamide, Irinotecan, and Radiation, in Patients With High‐Risk Rhabdomyosarcoma: A Report From the Children's Oncology Group,” J Clin Oncol 10, no. 2 (2016): 117–122, 10.1200/JCO.2015.63.4048. PMC5070550 26503200

[14] C. A. Arndt , J. A. Stoner , D. S. Hawkins , et al., “Vincristine, Actinomycin, and Cyclophosphamide Compared With Vincristine, Actinomycin, and Cyclophosphamide Alternating With Vincristine, Topotecan, and Cyclophosphamide for Intermediate‐Risk Rhabdomyosarcoma: Children's Oncology Group Study D9803,” J Clin Oncol 1, no. 31 (2009): 5182–5188, 10.1200/JCO.2009.22.3768. PMC2773476 19770373

[15] M. Carli , R. Colombatti , O. Oberlin , et al., “European Intergroup Studies (MMT4‐89 and MMT4‐91) on Childhood Metastatic Rhabdomyosarcoma: Final Results and Analysis of Prognostic Factors,” J Clin Oncol 22, no. 23 (2004): 4787–4794, 10.1200/JCO.2004.04.083. 15570080

[16] T. Klingebiel , J. Boos , F. Beske , et al., “Treatment of Children With Metastatic Soft Tissue Sarcoma With Oral Maintenance Compared to High Dose Chemotherapy: Report of the HD CWS‐96 Trial,” Pediatr Blood Cancer 50, no. 4 (2008): 739–745, 10.1002/pbc.21494 18286501

[17] L. Tramsen , K. Bochennek , M. Sparber‐Sauer , et al., “Pediatric Patients With Stage IV Rhabdomyosarcoma Significantly Benefit From Long‐Term Maintenance Therapy: Results of the CWS‐IV 2002 and the CWS DOK IV 2004‐Trials,” Cancers (Basel) 15, no. 7 (2023): 2050, 10.3390/cancers15072050. 37046711 PMC10093505

[18] H. P. McDowell , A. B. Foot , C. Ellershaw , D. Machin , C. Giraud , and C. Bergeron , “Outcomes in Paediatric Metastatic Rhabdomyosarcoma: Results of the International Society of Paediatric Oncology (SIOP) Study MMT‐98,” Eur J Cancer 46, no. 9 (2010): 1588–1595, 10.1016/j.ejca.2010.02.051. 20338746

[19] G. Bisogno , A. Ferrari , A. Prete , et al., “Sequential High‐Dose Chemotherapy for Children With Metastatic Rhabdomyosarcoma,” Eur J Cancer 45, no. 17 (2009): 3035–3041, 10.1016/j.ejca.2009.08.019. 19783136

[20] S. Fetzko , A. A. Gupta , B. A. Setty , et al., “Salvage Therapy Efficacy Is Modified by Risk Group at Diagnosis in Patients With Relapsed Rhabdomyosarcoma,” Pediatr Blood Cancer 72, no. 3 (2025): e31477, 10.1002/pbc.31477. 39654081

[21] A. C. Mattke , E. J. Bailey , A. Schuck , et al., “Does the Time‐Point of Relapse Influence Outcome in Pediatric Rhabdomyosarcomas?,” Pediatr Blood Cancer 52, no. 7 (2009): 772–776, 10.1002/pbc.21906 19165889

[22] L. Mascarenhas , E. R. Lyden , P. P. Breitfeld , et al., “Randomized Phase II Window Trial of Two Schedules of Irinotecan With Vincristine in Patients With First Relapse or Progression of Rhabdomyosarcoma: A Report From the Children's Oncology Group,” J Clin Oncol 28, no. 30 (2010): 4658–4663, 10.1200/JCO.2010.29.7390. 20837952 PMC2974343

[23] L. Mascarenhas , Y. Y. Chi , P. Hingorani , et al., “Randomized Phase II Trial of Bevacizumab or Temsirolimus in Combination With Chemotherapy for First Relapse Rhabdomyosarcoma: A Report From the Children's Oncology Group,” J Clin Oncol 37, no. 31 (2019): 2866–2874, 10.1200/JCO.19.00576. 31513481 PMC6823886

[24] A. S. Defachelles , E. Bogart , M. Casanova , et al., “Randomized Phase II Trial of Vincristine‐Irinotecan With or Without Temozolomide, in Children and Adults With Relapsed or Refractory Rhabdomyosarcoma: A European Paediatric Soft Tissue Sarcoma Study Group and Innovative Therapies for Children With Cancer Trial,” J Clin Oncol 39, no. 27 (2021): 2979–2990, 10.1200/JCO.21.00124. 34343032

[25] J. Chisholm , H. Mandeville , M. Adams , et al., “Frontline and Relapsed Rhabdomyosarcoma (FAR‐RMS) Clinical Trial: A Report From the European Paediatric Soft Tissue Sarcoma Study Group (EpSSG),” Cancer 16 (2024): 998, 10.3390/cancers16050998. PMC10931395 38473359

