# Supplementary material for: Children and Young People With First Relapse or Progression of Upfront Metastatic Rhabdomyosarcoma: An Analysis of Clinical Features and Outcomes From the INternational Soft Tissue saRcoma ConsorTium (INSTRuCT)
Source: Cancer Med. 2026 Feb 20;15(3):e71524. doi: 10.1002/cam4.71524 (PMC12927941; doi:10.1002/cam4.71524)
Supplement: Supplementary file 1 — Table S1. Upfront Cohort: Studies list by data contributor. Table S2. Studies list by data contributor in First Event Cohort. Table S3. Univariate analysis of Overall Survival from first event in First Event Cohort (n = 637*). [file CAM4-15-e71524-s001.docx]

**Supplementary Table 1:Upfront Cohort: Studies list by data contributor**

|  | **COG**  **N (%)** | **EpSSG**  **N (%)** | **CWS**  **N (%)** | **MMT**  **N (%)** | **ICG**  **N (%)** | **Total (%)** |
| --- | --- | --- | --- | --- | --- | --- |
| **Study** |  |  |  |  |  |  |
| MTS2008 | - | 262 (100) | - | - | - | 262 (23.9) |
| ARST08P1 | 157 (38.7) | - | - | - | - | 157 (14.3) |
| MMT 98 | - | - | - | 106 (100) | - | 106 (9.7) |
| CWS-96 HD | - | - | 110 (43.7) | - | - | 110 (10.1) |
| D9802 | 106 (26.1) | - | - | - | - | 106 (9.7) |
| ARST0431 | 99 (24.4) | - | - | - | - | 99 (9.0) |
| CWS-DOK IV 2004 | - | - | 84 (33.3) | - | - | 84 (7.7) |
| RMS4.99 | - | - | - | - | 69 (100) | 69 (6.3) |
| D9803 | 44 (10.8) | - | - | - | - | 44 (4.0) |
| CWS-91 | - | - | 34 (13.5) | - | - | 34 (3.1) |
| CWS-IV-2002 | - | - | 24 (9.5) | - | - | 24 (2.2) |
| **Total** | **406 (37.1)** | **262 (23.9)** | **252 (23.0)** | **106 (9.7)** | **69 (6.3)** | **1095 (100)** |

**Supplementary Table 2: Studies list by data contributor in First Event Cohort**

|  | **COG**  **N (%)** | **EpSSG**  **N (%)** | **CWS**  **N (%)** | **MMT**  **N (%)** | **ICG**  **N (%)** | **Total (%)** |
| --- | --- | --- | --- | --- | --- | --- |
| **Study** |  |  |  |  |  |  |
| MTS2008 | - | 165 (100) | - | - | - | 165 (22.7) |
| ARST08P1 | 119 (41.3) | - | - | - | - | 119 (16.4) |
| MMT 98 | - | - | - | 53 (100) | - | 53 (7.3) |
| CWS-96 HD | - | - | 73 (41.5) | - | - | 73 (10.0) |
| D9802 | 88 (30.6) | - | - | - | - | 88 (12.1) |
| ARST0431 | 61 (21.2) | - | - | - | - | 61 (8.4) |
| CWS DOK IV 2004 | - | - | 60 (34.1) | - | - | 60 (8.3) |
| RMS4.99 | - | - | - | - | 45 (100) | 45 (6.2) |
| D9803 | 20 (6.9) | - | - | - | - | 20 (2.8) |
| CWS-91 | - | - | 25 (14.2) | - | - | 25 (3.4) |
| CWS-IV-2002 | - | - | 18 (10.2) | - | - | 18 (2.5) |
| **Total** | **288 (39.6)** | **165 (22.7)** | **176 (24.2)** | **53 (7.3)** | **45 (6.2)** | **727 (100)** |

**Supplementary Table 3: Univariate analysis of Overall Survival from first event in First Event Cohort (n = 637*)**

|  | **3-yr OS (95%CI)** | **p-value** |
| --- | --- | --- |
| **Sex** |  |  |
| Female | 6.9 (4.3-10.3) | 0.6129 |
| Male | 8.9 (6.1-12.2) |  |
| **Age at diagnosis** |  |  |
| <1 or ≥10 years | 6.0 (7.8-16.3) | **0.0354** |
| 1-9 years | 11.6 (7.8-16.3) |  |
| **Histology** |  |  |
| Favourable RMS | 9.3 (5.6-14.3) | 0.3790 |
| Unfavourable RMS | 7.4 (5.2-10.1) |  |
| **Tumour site** |  |  |
| Favourable | 8.3 (5.0-12.8) | 0.4876 |
| Unfavourable | 7.8 (5.4-10.6) |  |
| **Tumour size** |  |  |
| ≤5 cm | 9.9 (5.7-15.4) | 0.8945 |
| >5 cm | 7.4 (5.2-10.0) |  |
| **T-invasiveness** |  |  |
| T1 | 12.9 (6.7-21.3) | **0.0451** |
| T2 | 7.2 (5.2-9.6) |  |
| **Nodal involvement** |  |  |
| N0 | 12.6 (8.6-17.3) | **0.0007** |
| N1 | 5.4 (3.4-7.9) |  |
| **Number of metastatic sites** |  |  |
| 1-2 sites | 10.8 (8.0-14.0) | **<0.0001** |
| ≥ 3 sites | 2.1 (0.7-4.9) |  |
| **Oberlin score** |  |  |
| 0-1 factors | 16.6 (11.5-22.5) | **<0.0001** |
| ≥ 2 factors | 4.6 (2.9-6.9) |  |
| **Type of first event** |  |  |
| Local/Loco-regional relapse | 13.6 (7.8-21.1) | **<0.0001** |
| Metastatic relapse | 7.6 (5.1-10.7) |  |
| Progressive disease | 5.3 (2.5-9.7) |  |
| **Time to first event** |  |  |
| ≤18 months | 3.2 (1.8-5.2) | **<0.0001** |
| >18 months | 17.8 (12.8-23.4) |  |

*Ninety patients with one or more of uncertain nodal involvement , T status, tumour size or number of metastatic sites were excluded from this analysis.
